# Identification of Candidate Biomarker ASXL2 and Its Predictive Value in Pancreatic Carcinoma

**DOI:** 10.3389/fonc.2021.736694

**Published:** 2021-10-08

**Authors:** Gaoming Wang, Ludi Yang, Jinli Gao, Huiling Mu, Yanxiang Song, Xiaohua Jiang, Bo Chen, Ran Cui

**Affiliations:** ^1^ Department of Hepatopancreatobiliary Surgery, Shanghai East Hospital, School of Medicine, Tongji University, Shanghai, China; ^2^ Department of Ophthalmology, Ninth People’s Hospital, Shanghai Jiao Tong University School of Medicine, Shanghai, China; ^3^ Department of Pathology, Shanghai East Hospital, School of Medicine, Tongji University, Shanghai, China

**Keywords:** ASXL2, prognosis, immunotherapy, pancreatic cancer, biomarker

## Abstract

Pancreatic adenocarcinoma is one of the most lethal diseases with a 5-year survival rate of about 8%. ASXL2 is an epigenetic regulator associated with various tumors including colorectal cancer, breast cancer, and myeloid leukemia. However, the role of ASXL2 in pancreatic cancer remains unclear. This is the first research focusing on the prognostic value of ASXL2 in pancreatic cancer. In this research, we aimed to explore the correlation between ASXL2 and the prognosis, as well as other features in PAAD. We obtained gene expression profiles of PAAD and normal tissues from TCGA, GEO, and Xena databases. TIMER and CIBERSORT algorithms were employed to investigate the effect of ASXL2 on tumor microenvironment. GSEA along with GO and KEGG enrichment analyses were conducted to uncover the biological functions of ASXL2. The response to various chemotherapeutic drugs was estimated by algorithms in R package “pRRophetic”, while the sensitivity to immunotherapy was quantified by TIDE score. We found that ASXL2 was upregulated in the PAAD samples and elevated expression of ASXL2 was linked to poor overall survival. ASXL2 DNA methylation contributed to ASXL2 expression. Functional annotation indicated that ASXL2 was mainly involved in inflammatory response and epithelial mesenchymal transition. Patients with high ASXL2 expression were more likely to benefit from immune checkpoint blockade, gemcitabine, and mitomycin-C. Finally, external datasets and biospecimens were used and the results further validated the aberrant expression of ASXL2 in PAAD samples. In summary, our results highlight that ASXL2 is a potential prognostic and predictive biomarker in pancreatic cancer.

## Introduction

Pancreatic adenocarcinoma (PAAD) is a predominant part of pancreatic cancer, which is one of the most lethal malignancies. With various advances in surgical technique and imaging technology, patients would receive surgical resection if the tumor was localized to the pancreas. However, 80-85% of patients are diagnosed with advanced disease and most treatments are ineffective, which contributes to the poor prognosis of PAAD with a 5-year survival rate of only about 8% (1–3). What’s worse, the effect of chemotherapy varies from person to person because of the development of chemoresistance (4). Meanwhile, the efficacy of immunotherapeutic strategies is under assessment and there has no study that exhibited practice changing results (5). Recently, molecular biomarkers have been demonstrated to be helpful in the diagnosis and prognosis of diseases. Therefore, the discovery of new molecular biomarkers for predicting prognosis and PAAD therapy is urgently needed.

ASXL2 encodes a protein serving as an epigenetic regulator and is associated with various types of cancer. An increasing number of studies have suggested that ASXL2 is an invasion-driver gene and overexpression of ASXL2 is correlated with prognosis in colorectal cancer (6, 7). In breast cancer, ASXL2 is demonstrated to promote tumor proliferation through linking ERalpha to histone methylation (8). While ASXL2 is identified to be essential for hematopoiesis and loss of Asxl2 in mice can lead to myeloid malignancies (9, 10). Collectively, these studies highlight that ASXL2 may play a dual role in tumorigenesis and development. Although pancreatic cancer is one of the common malignancies, the role of ASXL2 in pancreatic cancer remains undefined.

In this study, we aimed to explore the prognostic value of ASXL2 in PAAD. We investigated the expression of ASXL2 in human PAAD samples using RNA-seq and microarray data obtained from TCGA and GEO databases, respectively. The correlation between ASXL2 expression and the prognosis in PAAD patients, as well as clinical features, were analyzed. As tumor immunogenicity and immune cells may be affected by epigenomic alterations, we tried to explore the roles of ASXL2 in tumor immune microenvironment (TIME) (11). To better understand the biological functions of ASXL2 in PAAD, we conducted Gene Ontology (GO) and Kyoto Encyclopedia of Genes and Genomes (KEGG) analyses along with Gene Set Enrichment Analysis (GSEA). In addition, we got an insight into the correlation between ASXL2 expression and the response to chemotherapy and immunotherapy. Finally, the aberrant expression of ASXL2 in pancreatic cancer was validated by external datasets and biospecimens.

As the first study concentrating on ASXL2 and pancreatic cancer, our work suggested that ASXL2 expression is correlated with the OS among PAAD patients, several tumor-infiltrating immune cells, and the possible response to chemotherapy and immunotherapy, which highlight the prognostic and predictive value of ASXL2 in pancreatic cancer.

## Materials and Methods

### Data Collection

The gene expression profiles of PAAD samples and normal tissues were obtained from The Cancer Genome Atlas (TCGA) database (TCGA-PAAD, https://portal.gdc.cancer.gov/), the UCSC Xena project (http://xena.ucsc.edu/), and Gene Expression Omnibus (GEO) database (GSE28735 (12), GSE62452 (13), https://www.ncbi.nlm.nih.gov/geo/). The Xena project has recomputed all expression raw data from TCGA and GTEx (the Genotype-Tissue Expression, https://www.gtexportal.org/home/) based on a standard pipeline to minimize differences from distinct sources. The corresponding clinical information of PAAD patients from TCGA and methylation data were downloaded from the cBioPortal website (14) (https://www.cbioportal.org/). A total of 292 PAAD samples (TCGA: 178; GSE28735: 45; GSE62452: 69) and 277 normal tissues (TCGA: 4; GTEx: 167; GSE28735: 45; GSE62452: 61) were enrolled in this study.

### Correlation Between ASXL2 and Tumor-Infiltrating Immune Cells

Tumor Immune Estimation Resource (TIMER, http://timer.cistrome.org/) is a comprehensive resource for systematical analysis of immune infiltrates across diverse cancer types (15). By taking advantage of TIMER, we assessed the relationship between ASXL2 and the abundance of six types of immune infiltrating cells (B cells, CD8+ T cells, CD4+ T cells, macrophages, neutrophils, and dendritic cells) in PAAD.

In addition, CIBERSORT algorithm was utilized to evaluate the fractions of 22 types of tumor-infiltrating cells to further confirm the effect of ASXL2 on tumor immune microenvironment (TIME) in PAAD (16). Only samples with P < 0.05 were included for further study.

### Functional Enrichment Analyses

Differentially expressed genes (DEGs) between two groups were screened by using “DESeq2” package in R software according to the thresholds of |log2FoldChange| > 1 and adjusted p-value (padj) < 0.05 (17). Gene Ontology (GO) and Kyoto Encyclopedia of Genes and Genomes (KEGG) enrichment analyses were performed to annotate the biological functions of DEGs and ASXL2-related genes through “clusterProfiler” package (18). With the annotated gene sets in “h.all.v7.4.symbols.gmt” chosen as the reference gene sets, gene set enrichment analysis (GSEA) was conducted to investigate the potential regulatory mechanisms of ASXL2 (18, 19).

### Estimation of the Sensitivity to Chemotherapy and Immunotherapy

We estimated the IC_50_ of common chemotherapeutic drugs *via* “pRRophetic” package (20). The potential response to immunotherapy was predicted and qualified by Tumor Immune Dysfunction and Exclusion (TIDE) algorithm *via* python (version 3.8.6) language (21). The lower TIDE score, the more sensitive to immune checkpoint blockade (ICB).

### Biospecimens and Immunohistochemistry

Biospecimens histopathologically confirmed PAAD were obtained from Shanghai East Hospital Biobank. A total of 10 PAAD samples and 5 para-cancerous tissues were collected for immunohistochemistry. All patients had signed informed consent for donating their specimens to Shanghai East Hospital Biobank. Biopsy samples were fixed with formalin followed by paraffin embedding. Immunohistochemistry was conducted using anti-ASXL2 antibody (1:200, Abcam). Images of slides were taken by a Leica microscope equipped with a digital camera at 10× and 10× magnification. Staining was independently evaluated by two experienced pathologists blinded to the clinical information of patients. The score for ASXL2 staining was based on the staining intensity and the percentage of positive cells. Staining intensity was quantified as follows: 0: no color; 1: yellow; 2: light brown; and 3: dark brown. The percentage of positive cells were scored as followings: 0: positive cells < 5%; 1: 6%- 25%; 2: 26%- 50%; 3: 51%- 75%; and 4: > 75%. The staining score was calculated as the score of staining intensity × the score of percentage of positive cells.

### Statistical Analysis

All statistical tests were conducted *via* R (version 4.0.3) software. Comparisons between two groups were conducted with Wilcoxon rank-sum test. Correlation analysis was performed using Pearson correlation test. Categorical variables between groups were compared with chi-square test. Kaplan-Meier survival curves were plotted to exhibit the overall survival (OS) for PAAD patients. Univariate and multivariate Cox regression analyses were employed to determine the independent prognostic significance of each variable enrolled in this study. P < 0.05 was considered statistically significant.

## Results

### The Aberrant Expression and Prognostic Value of ASXL2 in PAAD Patients

**Figure 1:**
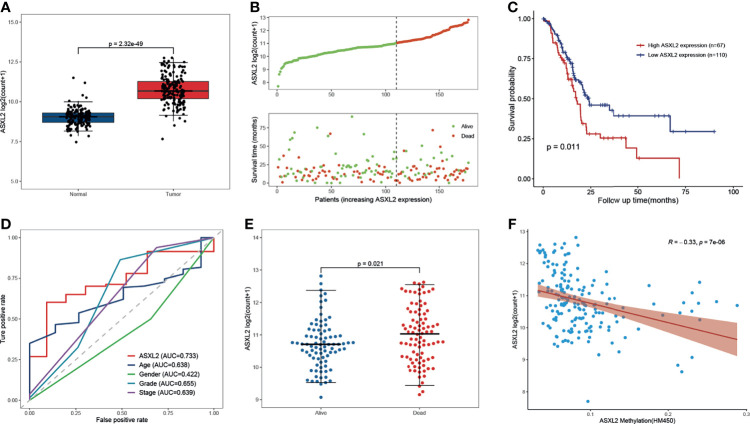
The abnormal expression and prognostic value of ASXL2 in PAAD. **(A)** Box plot of ASXL2 expression in normal and PAAD tissues. **(B)** Distribution of ASXL2 expression, survival time, and survival statuses of PAAD patients. The dotted line represents the cut-off value. **(C)** Kaplan-Meier survival analyses of ASXL2 in PAAD patients on overall survival. **(D)** ROC curve analysis to evaluate the prognostic value of ASXL2 expression and clinical factors in PAAD at 5-year-survival. **(E)** The correlation between ASXL2 expression and survival status. **(F)** The relationship between ASXL2 expression and its DNA methylation.

The RNA-seq data of 178 PAAD samples along with 4 adjacent normal tissues from TCGA and 167 normal samples from the GTEx were analyzed, and we found that the mRNA expression level of ASXL2 was distinctly high in PAAD samples (*P* = 1.31e-23, **Figure 1A**). To investigate the prognostic significance of ASXL2 in PAAD patients, we divided the patients into high and low ASXL2 expression groups based on the optimal cutoff calculated *via* “survival” and “survminer” packages (**Supplementary Figure 1A**). The distribution of ASXL2 expression and survival status of PAAD patients were exhibited in **Figure 1B**. Kaplan-Meier survival curve demonstrated that PAAD patients with low ASXL2 expression levels showed longer survival time (*P* = 0.011, **Figure 1C**). The ROC curve showed that ASXL2 expression had a certain power for predicting the 5-year-OS and PAAD patients with high ASXL2 expression tended to exhibit worse clinical outcomes (**Figures 1D, E**). Then, we investigate the correlation between ASXL2 expression and clinicopathological factors using logistic regression analysis and found that elevated ASXL2 expression levels in PAAD were correlated with tumor grade (G4 vs G1, *P* = 0.002) and ASXL2 DNA methylation (*P* < 0.001, **Table 1**). In addition, patients with PAAD were divided into high or low ASXL2 expression subgroups according to the median value and the chi-square test was conducted to further study the relationships between ASXL2 expression and clinical features. The results indicated that ASXL2 expression was significantly correlated with survival status (*P* = 0.049) and ASXL2 DNA methylation (*P* < 0.001, **Supplementary Table 1**). As shown in **Figure 1F**, the expression level of ASXL2 was negatively correlated with the methylation level (*R* = -0.33, *P* = 7e-06, **Figure 1F**). Taken together, ASXL2 may be a potential prognostic biomarker for PAAD patients.

**Table 1:** Association between ASXL2 expression and clinical features using logistic regression.

Clinical characteristic	OR	CI	P-value
Age	0.992	0.98-1.003	0.145
Gender (Male vs Female)	0.873	0.682-1.118	0.283
Grade (G2 vs G1)	1.175	0.833-1.657	0.36
Grade (G3 vs G1)	1.407	0.957-2.069	0.084
Grade (G4 vs G1)	0.151	0.047-0.483	**0.002**
Grade (Gx vs G1)	1.169	0.36-3.801	0.795
Stage (II vs I)	1.081	0.738-1.584	0.689
Stage (III vs I)	0.841	0.314-2.254	0.731
Stage (IV vs I)	1.594	0.663-3.832	0.299
ASXL2 DNA methylation	0.003	0-0.032	**0**

The bold values mean that P-value < 0.05.

### The Correlation Between ASXL2 and Tumor-Infiltrating Immune Cells

**Figure 2:**
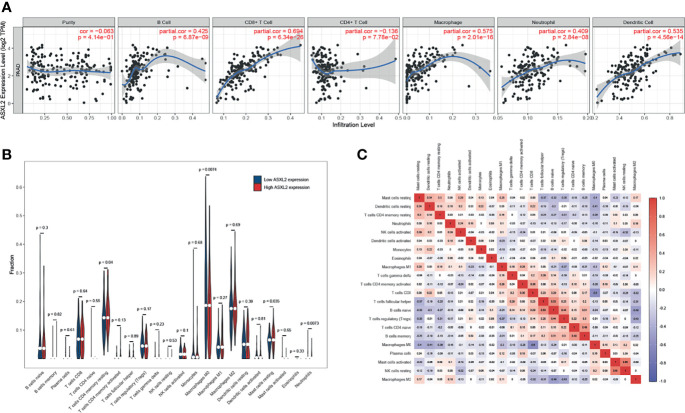
The effect of ASXL2 on tumor immune microenvironment. **(A)** The correlation between ASXL2 and various types of tumor-infiltrating immune cells. **(B)** The infiltrating levels of 22 types of immune cells in high and low ASXL2 expression groups. **(C)** Heatmap of 22 types of immune cells in PAAD.

To explore the effect of ASXL2 on TIME, we took advantage of TIMER website. As shown in **Figure 2A**, ASXL2 expression exhibited a positive correlation with the fractions of B cells (*P* = 6.87e-09), CD8+ T cells (*P* = 6.34e-26), macrophages (*P* = 2.01e-16), neutrophils (*P* = 2.84e-08), and dendritic cells (*P* = 4.56e-14). What’s more, to further confirm the role of ASXL2, we employed CIBERSORT algorithm to estimate the levels of 22 types of immune cells. After removing PAAD samples with *P* ≥ 0.05, a total of 124 samples remained and were assigned into a high or low ASXL2 expression group based on the median value. When compared to samples with low ASXL2 expression, the proportions of T cells CD4 memory resting, mast cells resting, and neutrophils were distinctly increased, however, macrophages M0 in PAAD decreased (**Figure 2B** and **Supplementary Figure 2**). Relationships between 22 kinds of immune cells indicated that T cells CD4 memory resting and mast cells resting were weakly positively correlated and both of them were negatively correlated with macrophages M0 (**Figure 2C**). These findings suggested that ASXL2 played a crucial role in immune infiltration in PAAD.

### The Biological Functions of ASXL2 in PAAD

**Figure 3:**
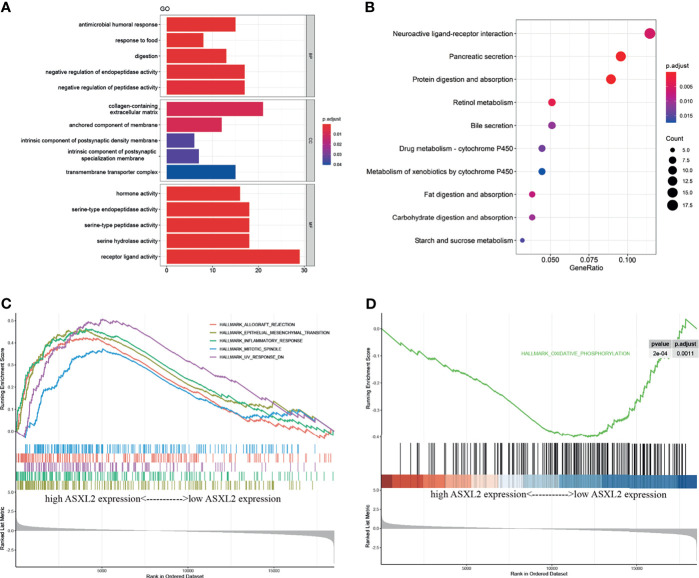
Functional annotation of ASXL2. **(A)** GO enrichment analyses of DEGs based on ASXL2 expression. BP, biological process; CC, cellular component; MF, molecular function. **(B)** KEGG enrichment analysis of DEGs based on ASXL2 expression in PAAD. **(C, D)** GSEA analyses to explore the potential regulatory mechanisms with hallmarks set as reference gene sets.

Since ASXL2 contributes to the TIME, we next got an insight into the potential mechanisms underlying ASXL2. A total of 387 differentially expressed genes (DEGs) between low and high ASXL2 expression groups were screened. Among these DEGs, 123 were upregulated, while 264 were downregulated in the high ASXL2 expression group (**Supplementary Figure 1B** and **Supplementary Table 2**). Functional annotation including GO term and KEGG pathway analyses of these DEGs were conducted. GO annotation indicated that ASXL2-associated DEGs were mainly involved in processes like “negative regulation of peptidase activity” and “receptor ligand activity” (**Figure 3A**). Meanwhile, KEGG pathway analysis showed that pathways including “neuroactive ligand-receptor interaction”, “pancreatic secretion”, and “protein digestion and absorption” were significantly enriched (**Figure 3B**). As the expression level of ASXL2 was correlated with tumor grade and the prognosis of PAAD patients, we hypothesized that increased expression of ASXL2 promotes tumor progression. We employed GSEA and found that hallmarks of tumor such as “epithelial mesenchymal transition”, “inflammatory response” and “mitotic spindle” were dynamically correlated with the high ASXL2 expression, while “oxidative phosphorylation” was significantly enriched in the low ASXL2 expression group (**Figures 3C, D** and **Supplementary Table 2**).

### ASXL2 Could Serve as a Biomarker to Predict the Response to Chemotherapy and ICB

**Figure 4:**
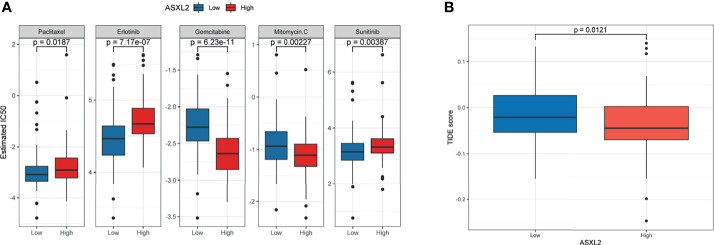
Evaluate the predictive value of ASXL2 in clinical treatment. **(A)** The estimated IC_50_ of common chemotherapeutic drugs. **(B)** TIDE score of different ASXL2 expression groups.

Adjuvant chemotherapy can distinctly improve the survival rate of patients with curative resection of pancreatic cancer (22). Drugs including paclitaxel, erlotinib, gemcitabine, mitomycin C, and sunitinib are demonstrated to be helpful in pancreatic cancer treatment (23–26). On the other hand, the efficacy of immunotherapeutic strategies is under assessment. To figure out the potential role of ASXL2 in clinical treatment decisions, we estimated the IC_50_ of common drugs recommended for pancreatic cancer therapy by AJCC guidelines. Interestingly, patients in the low ASXL2 expression group tended to be more sensitive to chemotherapeutic drugs such as paclitaxel, erlotinib, and sunitinib, while patients with high ASXL2 expression were more likely to benefit from gemcitabine and mitomycin C (**Figure 4A**). Interactions between tumor cells and tumor-infiltrating immune cells affect the effect of ICB (21, 27). Based on the therapy, we calculated the TIDE score of each sample using gene expression profiles. The results showed that the level of TIDE score in the high ASXL2 expression group was lower than that in the low ASXL2 expression group, which indicated that PAAD patients with high ASXL2 expression levels were more likely to respond to ICB (**Figure 4B**). Our results suggested that ASXL2 may help therapeutic strategies making for PAAD patients.

### Validation of the Elevated Expression of ASXL2 in External Datasets and Biospecimens

**Figure 5:**
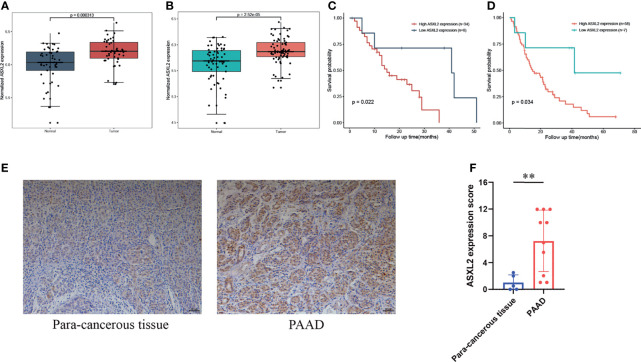
Validation of the elevated expression and prognostic value of ASXL2. **(A, B)** ASXL2 was highly expressed in pancreatic tumor tissues, and data were obtained from GSE28735 **(A)** and GSE62452 **(B)**. **(C, D)** Patients with high ASXL2 showed poor overall survival, and data were extracted from GSE28735 **(C)** and GSE62452 **(D)**. **(E)** Representative immunohistochemistry staining of ASXL2 in PAAD samples and para-cancerous tissues. **(F)** Quantified data of the score for ASXL2 staining. **P < 0.01.

To further validate the aberrant expression of ASXL2 in pancreatic cancer, we obtained additional gene expression data from GSE28735 and GSE62452. Compared with the adjacent normal tissues, ASXL2 was significantly upregulated in tumor samples (**Figures 5A, B**). In addition, Kaplan-Meier survival analyses showed that patients with low ASXL2 expression survival longer than those with high ASXL2 expression, which highlights the prognostic value of ASXL2 in PAAD (**Figures 5C, D**). We conducted immunohistochemistry staining of ASXL2 in PAAD tissues and adjacent normal tissues, and the results agreed with our findings (**Figures 5E, F**).

## Discussion

Pancreatic cancer is one of the deadly malignancies and the prognosis remains extremally poor. Epigenetic aberrations including DNA methylation, histone modification, nucleosome remodeling, and microRNA have been demonstrated to be crucial in tumorigenesis (28, 29). ASXL2 belongs to a family of epigenetic regulators that participate in various histone modifications. Previous researches indicated that ASXL2 promotes cancer cell proliferation and invasion in colorectal cancer (6, 7) and breast cancer (8), while ASXL2 is found to be essential for hematopoiesis and as a tumor suppressor in leukemia (9). However, the role of ASXL2 in pancreatic cancer remains unclear. In the present study, we identified ASXL2 as a potential prognostic and predictive biomarker for pancreatic cancer. We extracted gene expression profiles from TCGA and Xena databases and noted that ASXL2 was highly expressed in pancreatic tumors. We evaluated the prognostic value of ASXL2 and found that ASXL2 expression was significantly correlated with the OS and survival status of PAAD patients. PAAD patients with high ASXL2 expression were more likely to present a poor clinical outcome than those with low ASXL2 expression. We got an insight into the potential regulatory mechanisms of aberrant expression of ASXL2. In the consideration that ASXL2 is involved in epigenetic regulatory processes, we explored the correlation between ASXL2 expression and its DNA methylation level and identified that these two factors were significantly negatively correlated, which helps to uncover the regulatory mechanisms of ASXL2.

A growing number of studies have reported that epigenetic modulators regulate tumor immune microenvironment through various processes (30, 31). Therefore, we investigated the effect of ASXL2 on the TIME in pancreatic cancer *via* several deconvolution algorithms. We found that ASXL2 expression was positively correlated with the fractions of mast cells resting, while negatively associated with macrophages M0. Mast cells are known to participate in a variety of processes including allergies, angiogenesis, and immune regulations (32). Mast cells are also found in tumor microenvironment and promote carcinogenesis by connecting with cancer cells (33, 34). Macrophages infiltrating into tumor microenvironment can provide trophic and nutritional support for tumor cells to promote disease progression, while they can also mediate antineoplastic effects through their phagocytic and oxidative functions (35). Antitumor M1-like and pro-tumor M2-like macrophage subpopulations in microenvironment exert opposing effects on tumor cells (36). Considering the influence of ASXL2 on tumor microenvironment, we can infer that elevated expression of ASXL2 promotes mast cells infiltration and contributes to the poor prognosis. Taken together, these findings suggest that ASXL2 may play a crucial role in the regulation of tumor microenvironment in PAAD.

Functional annotation including GO and KEGG enrichment analyses indicated that ASXL2 was mainly involved in regulation of peptidase activity and mediation of immune process. The results of GSEA revealed that several hallmarks of cancer including epithelial mesenchymal transition, inflammatory response, and mitotic spindle were significantly enriched in the high ASXL2 expression group. Epithelial mesenchymal transition is known to play crucial roles in carcinogenesis and progression *via* promoting cancer cells’ properties including mobility, invasion, and resistance to drugs (37–39). Inflammation is recognized as a hallmark of cancer because inflammatory cells and cytokines are more likely to contribute to tumor growth and progression than their antitumor response (40). We can infer that ASXL2 promotes tumor development *via* a variety of processes, leading to an unsatisfactory clinical outcome. However, further researches are required to investigate and validate the roles and regulatory mechanisms of ASXL2.

Chemotherapy can profoundly improve the prognosis of pancreatic cancer, while the development of chemoresistance is rising and varies from person to person (4). To evaluate the predictive value of ASXL2 in clinical treatment, we calculated the sensitivity to common chemotherapeutic drugs based on gene expression profiles. Our results indicated that patients in the low ASXL2 expression group tended to be more sensitive to paclitaxel, erlotinib, and sunitinib, while patients with high expression of ASXL2 were more likely to benefit from gemcitabine and mitomycin-C. The efficacy of drug combinations has been evaluated and some of them, such as gemcitabine combined with mitomycin-C, could significantly improve the prognosis in certain cancer (41). Immune checkpoint blockade (ICB) has exhibited great efficacy in several cancers including non-small cell lung cancer, melanoma, and renal cancer, while the role of ICB in pancreatic cancer is limited (42). Here, we employed TIDE, a computational method to predict ICB response by modeling two primary mechanisms of tumor immune evasion, to estimate the possible response to immunotherapy (21). Our results indicated that patients with high ASXL2 expression were more likely to benefit from ICB. Collectively, our findings suggest that ASXL2 may help therapeutic strategies decisions. However, the predictive value of ASXL2 is required to be further validated by clinical trials.

In conclusion, this is the first research to identify ASXL2 as a new potential prognostic biomarker in pancreatic cancer. Our work helps to uncover the roles of ASXL2 in tumor immune microenvironment, the development of pancreatic cancer, and its potential value in therapeutic strategies determination. With a better understanding of its biological functions and regulatory mechanisms, ASXL2 may contribute to making biomarker therapies more effective for pancreatic cancer treatment in the future.

## Data Availability Statement

The original contributions presented in the study are included in the article/**Supplementary Material**. Further inquiries can be directed to the corresponding authors.

## Author Contributions

GW, LY, and BC designed the study, analyzed data, and wrote the manuscript. XJ and RC provided funding acquisition. JG, HM, and YS performed the experiments and analyzed the data. BC, XJ, and RC supervised the research, analyzed data, and wrote the manuscript. All authors contributed to the article and approved the submitted version.

## Funding

The Outstanding Clinical Discipline Project of Shanghai Pudong (No. PWYgy2018- 02), Shanghai Science and Technology Committee (20Y11912100), The Research project of Shanghai Municipal Health Commission (20204Y0302) and The Research project of Pudong Science and Technology Commission (PKJ2020-Y20).

## Conflict of Interest

The authors declare that the research was conducted in the absence of any commercial or financial relationships that could be construed as a potential conflict of interest.

## Publisher’s Note

All claims expressed in this article are solely those of the authors and do not necessarily represent those of their affiliated organizations, or those of the publisher, the editors and the reviewers. Any product that may be evaluated in this article, or claim that may be made by its manufacturer, is not guaranteed or endorsed by the publisher.

## Supplementary Material

Supplementary Figure 1
**(A)** The optimal cutoff of ASXL2 expression in TCGA-PAAD. **(B)** DEGs based on the expression of ASXL2. **(C)** The optimal cutoff of ASXL2 expression in GSE28735. **(D)** The optimal cutoff of ASXL2 expression in GSE62452.

Supplementary Figure 2
**(A)** The correlation between ASXL2 expression and immune-infiltrating cells. **(B)** When compared to samples with low ASXL2 expression, the proportions of T cells CD4 memory resting, mast cells resting, and neutrophils were distinctly increased, however, macrophages M0 in PAAD decreased.

Supplementary Table 1 Correlation between ASXL2 mRNA expression/methylation and clinical characteristics in TCGA-PAAD.

Supplementary Table 2 The up- and down-regulated genes and enriched terms and pathways.
