# Supplementary figures and images for: Identification of Candidate Biomarker ASXL2 and Its Predictive Value in Pancreatic Carcinoma
Source: Front Oncol. 2021 Oct 8;11:736694. doi: 10.3389/fonc.2021.736694 (PMC8531590; doi:10.3389/fonc.2021.736694)

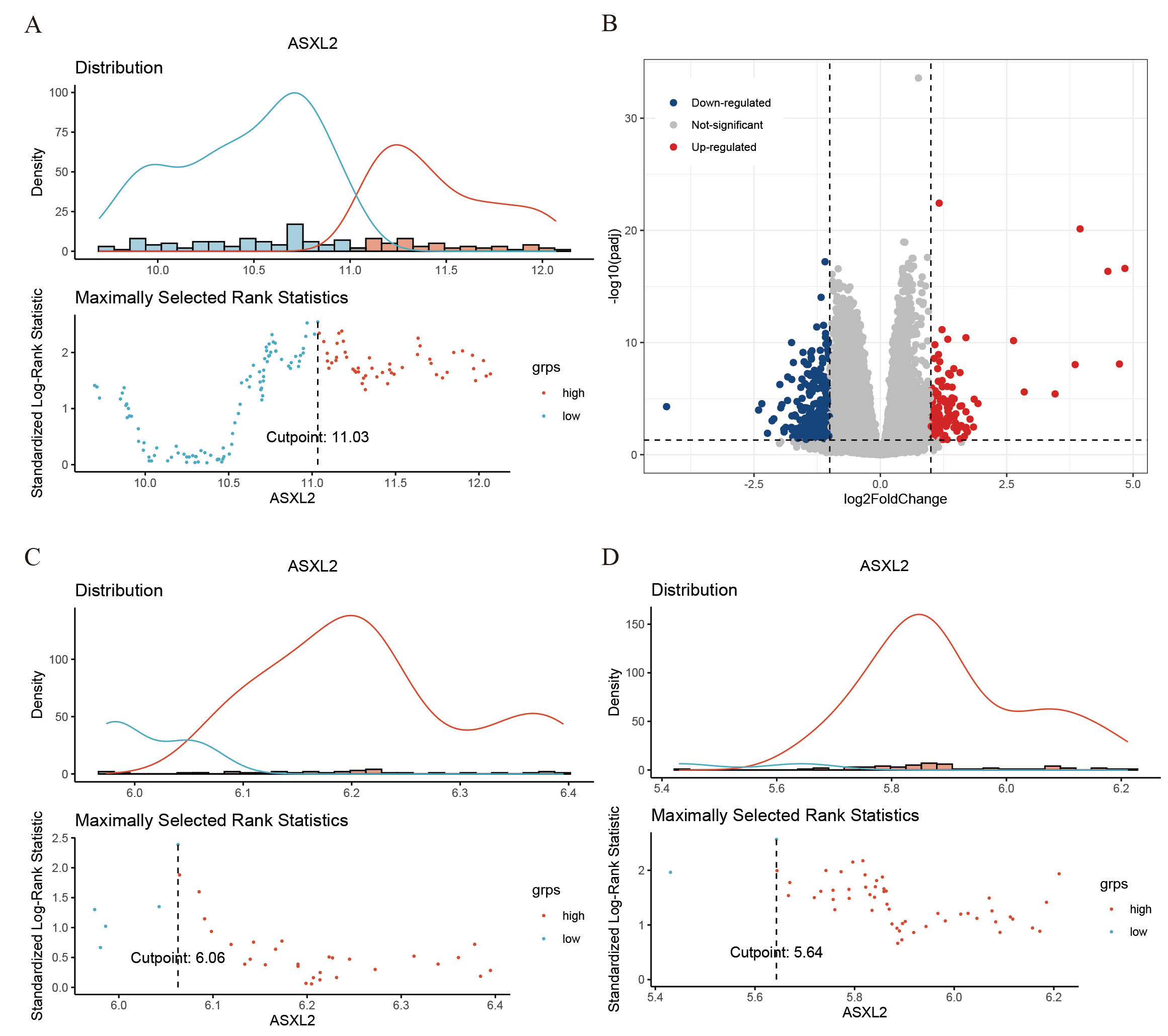

Supplement: Supplementary Figure 1 — (A) The optimal cutoff of ASXL2 expression in TCGA-PAAD. (B) DEGs based on the expression of ASXL2. (C) The optimal cutoff of ASXL2 expression in GSE28735. (D) The optimal cutoff of ASXL2 expression in GSE62452. [file Image_1.tif]

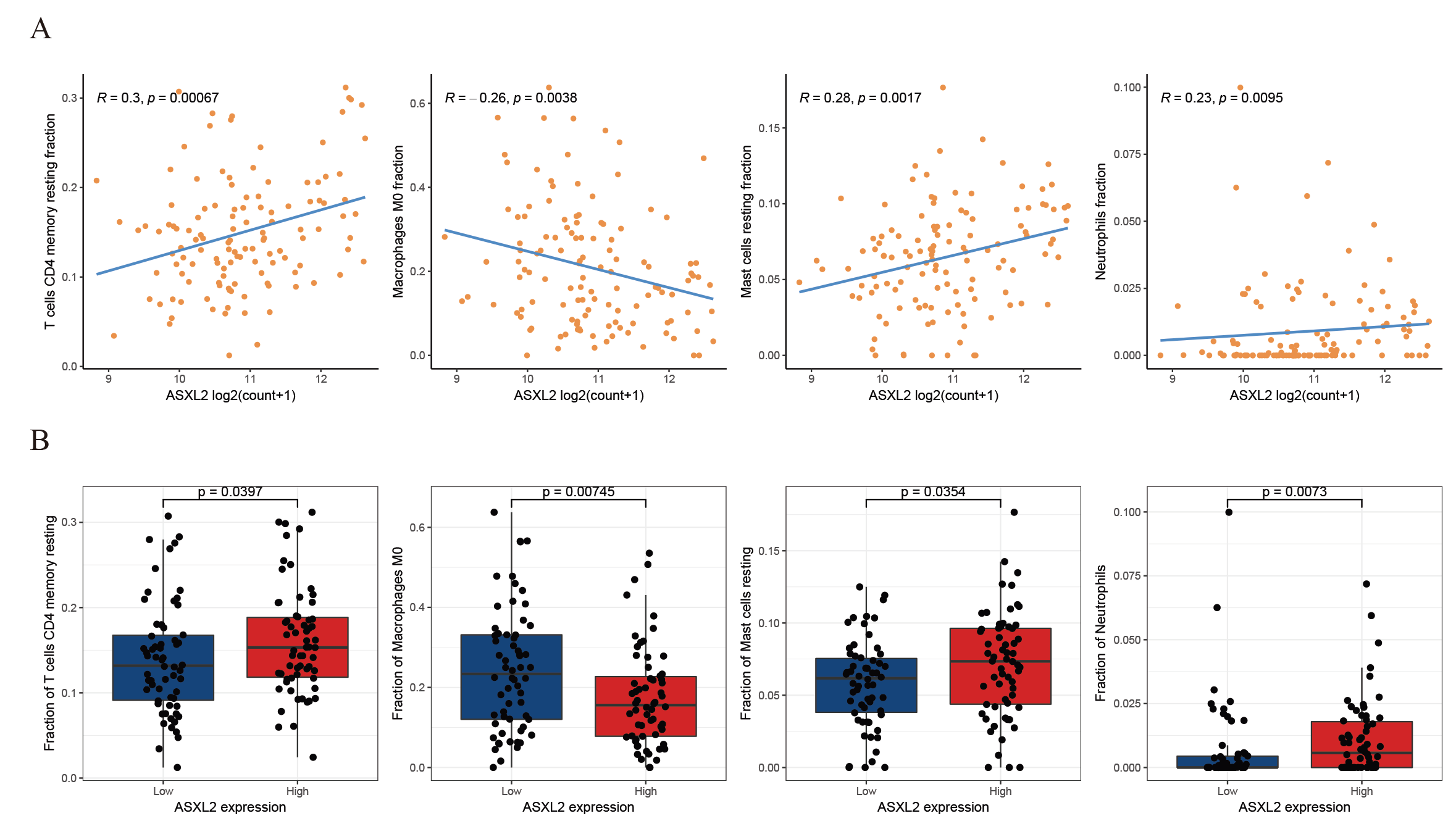

Supplement: Supplementary Figure 2 — (A) The correlation between ASXL2 expression and immune-infiltrating cells. (B) When compared to samples with low ASXL2 expression, the proportions of T cells CD4 memory resting, mast cells resting, and neutrophils were distinctly increased, however, macrophages M0 in PAAD decreased. [file Image_2.tif]
